# New Genus and Species of Webspinners (Insecta: Embioptera) from the Mid-Cretaceous of Myanmar with a Catalog of Fossil Members

**DOI:** 10.3390/insects15090636

**Published:** 2024-08-25

**Authors:** Siting Liu, Zihao Peng, Chaofan Shi, Dong Ren, Qiang Yang

**Affiliations:** 1 School of Life Sciences, Key Laboratory of Conservation and Application in Biodiversity of South China, Guangzhou University, #230 Waihuanxi Road, Guangzhou Higher Education Mega Center, Guangzhou 510006, China; lsting@e.gzhu.edu.cn (S.L.); 2112214031@e.gzhu.edu.cn (Z.P.); 2 School of Earth Sciences and Engineering, Guangdong Provincial Key Lab of Geological Processes and Mineral Resources, Sun Yat-sen University, Guangzhou 510275, China; 3 College of Life Sciences and Academy for Multidisciplinary Studies, Capital Normal University, Xisanhuanbeilu 105, Haidian District, Beijing 100048, China; rendong@mail.cnu.edu.cn

**Keywords:** Embioptera, Cretaceous, new genus, ocelli

## Abstract

**Simple Summary:**

There is an ongoing ambiguity surrounding the phylogenetic relationships of Embioptera, but fossil evidence can provide substantial information for phylogenetic analysis. Webspinners possess a potentially rich diversity within Cretaceous Myanmar amber. One new genus and three new species are described here and classified into the families Clothodidae and Scelembiidae. A comparison between extant and extinct species of the Clothodidae revealed that the non-cracking of the male tenth abdominal tergum, rather than hemitergites, may be a plesiomorphy. Additionally, the structure of the ocelli is increasingly found in fossil groups. These findings provide significant materials for studying the early evolution of Embioptera and understanding its morphology.

**Abstract:**

One new genus (*Ocrognethoda* gen. nov.) and three new species of webspinners (*Ocrognethoda olivea* gen. et sp. nov., *Gnethoda lata* sp. nov. and *Parasorellembia hamata* sp. nov.) are described from the Upper Cretaceous of Myanmar amber. *Ocrognethoda olivea* gen. et sp. nov. and *Gnethoda lata* sp. nov. are attributed to the family Clothodidae due to their simplified and symmetrical male terminalia, in which the tenth tergum is undivided. *Parasorellembia hamata* sp. nov. is classified in the family Scelembiidae by a specialized abdominal apex: fused cerci, a broad right hemitergite of the tenth abdominal tergite, and ocelli presence. Moreover, based on the discovery of new genus and species, the male terminalia of Clothodidae and Sorellembiinae are briefly discussed.

## 1. Introduction

Embioptera are a group that use their front feet to produce nanoscale silk for building shelters [1]. Usually, the silken galleries are constructed on trees or rocks, or amid leaf litter [2]. When confronted with noxious stimuli, webspinners are more inclined toward reverse movement [3]. The greatly enlarged femora of the hind legs are adapted to running backwards [2], and plenty of long setae in their sensitive cerci make them agile when engaging in backward movement [4].

So far, more than 400 extant webspinners and 28 fossil webspinners have been reported [5,6,7,8,9,10,11,12,13,14,15,16,17,18,19,20,21,22,23] (Table 1). The earliest fossil record of the webspinner dates back to the Permian–Triassic; they are considered to be the ancestors of the extant webspinners [8]. However, the specimens of this period only retain the structure of wings, and we lack knowledge of the body structure. Meanwhile, there are different views on whether two species—*Sinembia rossi* Huang and Nel, 2009 and *Juraembia ningchengensis* Huang and Nel, 2009—found in Inner Mongolia should be classified as Embioptera; Engel et al. suggested that they should be excluded from the order [21,24]. In recent years, as Myanmar amber has received much attention, 12 species of webspinners have been reported [11,12,13,14,15,21,22], and the family Clothodidae has the highest species richness.

Though the reconstruction of the phylogenetic relationships of Embioptera have been studied by some authors [25,26,27,28], the basal relationship of this taxon remains unclear. Most of the above phylogenetic studies are based on extant species and fossil members are rarely included, but fossil taxa may play an important role in reconstructing phylogenetic relationships in embiids [21]. Also, focusing on structures related to their lifestyle can provide useful phylogenetic information about several groups and sections of the tree [29].

Herein, we report one new genus and three new species from the Upper Cretaceous of northern Myanmar. The description of the new species can not only enrich the species record of the embiids but can also provide more material for clarifying the phylogenetic relationships.

## 2. Materials and Methods

This study is based on three specimens from Myanmar amber. The amber pieces were collected from the Hukawng Valley (the state of Kachin in northern Myanmar). The map of the Hukawng Valley was provided by Grimaldi et al. [30]. The volcaniclastic matrix of the amber is estimated to be approximately 98.79 ± 0.62 Ma, i.e., the earliest Cenomanian, near the Albian/Cenomanian (Early/Late Cretaceous) boundary [31]. The biological inclusions of Myanmar amber represent a sample of a tropical forest community in equatorial southeastern Asia at an approximately 12°N paleolatitude [30,32,33,34,35,36]. The specimens were permanently deposited in the collections of the Key Laboratory of Insect Evolution and Environmental Changes, College of Life Sciences, Capital Normal University, Beijing, China (CNUB; Dong Ren, Curator).

The specimens were examined using a Zeiss Discovery V20 stereomicroscope (Carl Zeiss, Oberkohen, Germany) and a Nikon SMZ1270 stereomicroscope (Nikon corporation, Tokyo, Japan) and photographed separately with an AxioCam HRc camera and an iMG SC600C digital camera attached to the Zeiss Discovery V20 (Carl Zeiss, Oberkohen, Germany) and Nikon SMZ1270 stereomicroscope (Nikon Corporation, Tokyo, Japan). The figures were processed using Adobe Illustrator CC 2018.

The morphological terminology in general follows Ross [2] and Engel et al. [21]. Abbreviations: A, analis; C, costa; CuA, anterior cubitus; CuP, posterior cubitus; MA, anterior media; MP, posterior media; R, radius; Rs, radial sector; Sc, subcostal. 10L, left hemitergite of tenth abdominal tergite; 10R, right hemitergite of tenth abdominal tergite; H, hypandrium; HP, hypandrial process; LC_1_, left basal cercomere; LC_2_, left apical cercomere; RC_1_, right basal cercomere; RC_2_, right apical cercomere; 10LP, left tergal process of tenth abdominal tergite; 10RP, right tergal process of tenth abdominal tergite; LPPT, process of left paraproct; and EP, epiproct.

## 3. Results

### Systematic Palaeontology

Order Embioptera Lameere, 1900.

Family Clothodidae Enderlein, 1909.

Subfamily Gnethodinae Cui and Engel, 2020.

Genus *Ocrognethoda* Liu, Shi, Ren and Yang gen. nov.

urn:lsid:zoobank.org:act:169BA2D5-D758-40B1-8A55-C5A4D48670C5

Type species. *Ocrognethoda olivea* Liu, Shi, Ren and Yang gen. et sp. nov.

Etymology. The generic name is a combination of the Greek *ocr*- (meaning “ridge”) and *Gnethoda* (the type genus of the family), referring to the middle of the caudal margin protruding in the tenth abdominal tergum. The gender is feminine.

Diagnosis. Elongated head; two plantulae (medial and apical plantulae) in hind metabasitarsus; tenth abdominal tergum with the middle of caudal margin protruded; hypandrium large, hypandrial process curved to the left; cerci nearly symmetrical, consist of two spindle segments; forewing with Sc terminating on anterior wing margin near basal two-fifths of wing length and connecting with anterior wing margin; MA not forked; CuA simple with one cua-cup crossvein; hind wing with CuA forked into CuA_1_ and CuA_2_.

Remarks. This genus can be assigned to the Clothodidae because of the following features: (1) symmetrical terminalia; (2) cylindrical cerci with two segments; and (3) tenth tergum is undivided. It can be classified as Gnethodinae because (1) forewing with MA and CuA simple; (2) metabasitarsus with medial and apical plantulae on ventral surface; and (3) left and right cerci identical and dimerous.

The new genus differs from other genera of this family by following characters: (1) forewing with MA not forked (MA forked in *Atmetoclothoda* Engel and Huang, 2016 and *Henoclothoda* Cui and Engel, 2020); (2) forewing with one cua-cup crossvein (no cua-cup crossvein present in *Genethoda* Cui and Engel, 2020); and (3) almost all longitudinal veins reaching wing margin (almost all longitudinal veins not reaching wing margin in *Perissoclothoda* Chen and Zhang, 2023).

*Ocrognethoda olivea* Liu, Shi, Ren and Yang gen. et sp. nov.

urn:lsid:zoobank.org:act:6D6B055F-CCB3-46E4-BC0C-D060CEC90B32

Figure 1.

Material. Holotype: CNU-EMB-MA2019024.

Etymology. The specific name is from the Latin *olivea* (meaning olive-shaped), referring to the olive-shaped head. The gender is feminine.

Locality and horizon. Hukawng Valley, Kachin State, northern Myanmar; lowermost Cenomanian, Upper Cretaceous.

Diagnosis. The same as for the genus.

**Figure 1:**
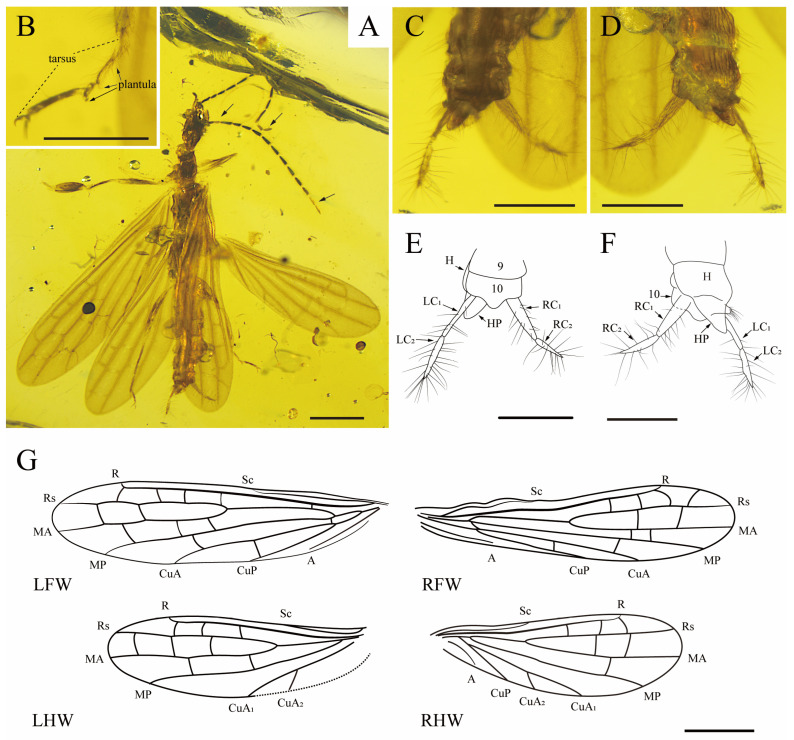
*Ocrognethoda olivea* gen. et sp. nov., holotype (CNU-EMB-MA2019024). (**A**) Habitus, dorsal view; (**B**) tarsus of right hind leg, ventral view; (**C**) photograph of male genitalia in dorsal view and (**D**) ventral view; (**E**) line drawing of male genitalia in dorsal view and (**F**) ventral view; (**G**) line drawing of wings, left forewing (LFW), right forewing (RFW), left hind wing (LHW), right hind wing (RHW). Scale bars: (**A**,**G**) 1 mm, (**B**–**F**) 0.5 mm.

Description. Male. Exoskeleton generally brown, with head dark-brown (Figure 1A). Body: total length (excluding wings, antennae, and cerci) ca. 5.70 mm. Head length (to apex of labrum) ca. 0.97 mm and width (just posterior to compound eyes) ca. 0.43 mm; compound eyes well-developed and prominent; labial palpus with three palpomeres, third palpomere longest; maxillary palpus with five palpomeres, apical palpomere longest; antenna preserved well, total length 3.43 mm with 12 flagellomeres—each flagellomeres with intensive setae, scape slightly longer than pedicel, and they are both longer than wide, first flagellomere length about five times as long as width, second to fourth flagellomeres length about three times as long as width, fifth through twelfth flagellomeres length about four times as long as width. Right pedicel and the last two flagellomeres yellow, other antennomeres dark brown. Pronotum length ca. 0.68 mm, width ca. 0.32 mm; anterolateral angles orthogonal; anterior margin weakly concave; posterior margin rounded; division into pro- and metazones distinct. Probasitarsus (first tarsal segment of fore leg) widened, with a length about three times as long as width (about 0.57 mm × 0.19 mm). Hind femora moderately widened; metabasitarsus (first tarsal segment of hind leg) with two plantulae (Figure 1B) located at the middle and apex of the segment; second tarsomere with one plantula. All claws simple and symmetrical, arolia absent. The ventral and dorsal plates of the abdomen cuticularized, and the margin of abdomen covered with elongated setae.

Terminalia (Figure 1C–F) nearly symmetrical; tenth abdominal tergum not divided into hemitergites, with the middle of caudal margin angularly protruded; hypandrial process (HP) broad and prominent, slightly sloping to the left; cerci nearly symmetrical, consisting of two spindle segments with abundant slender setae. Left basal cercal (LC_1_) length: ca. 0.34 mm, width: ca. 0.04 mm; left apical cercal (LC_2_) length: ca. 0.33 mm, width: ca. 0.04 mm; right basal cercal (RC_1_) length: ca. 0.33 mm, width ca. 0.08 mm; and right apical cercal (RC_2_) length: ca. 0.27 mm, width length: ca. 0.06 mm.

Forewing (Figure 1G): length ca. 4.65 mm, width ca. 1.21 mm, wings hyaline. Sc terminates on the anterior wing margin near the basal two-fifths of wing length and connects with anterior wing margin; R strong, connected with anterior wing margin, slightly procurved at the apex, with one faint r-rs+ma crossvein present in basal quarter of wing; Rs and MA separate near wing midlength; Rs simple, terminating on anterior wing margin apex, with four r-rs crossveins present in apical half of wing (three r-rs crossveins present and Rs bifurcate near wing apex in right forewing); MA simple, terminating at wing apex, with two rs-ma crossveins; MP simple, terminating on posterior wing margin, with three ma-mp crossveins (two ma-mp crossveins present in right forewing); CuA simple, terminating on posterior wing margin apical one-third of wing length, with two mp-cua crossveins, one present at anal area near the fork of CuA and CuP and the other present in apical one-third of wing length; CuP simple, terminating nearly at the wing midlength, with one cua-cup crossvein; A short and simple, not connected with posterior wing margin.

Hind wing (Figure 1G): length ca. 3.31 mm, width ca. 1.16 mm, wings hyaline. Sc terminates on the wing margin near the basal one-third of the wing length; R strong, connected with anterior wing margin, slightly procurved at the apex; Rs simple, terminating on anterior wing margin apex; three r-rs crossveins present in apical half of wing; MA simple, terminating at wing apex, with two rs-ma crossveins (three rs-ma crossveins present in left hind wing); MP simple, terminating on posterior wing margin, with one ma-mp crossvein (two ma-mp crossveins present in left hind wing); CuA divided into two branches at one-third of the wing length, with CuA_1_ terminating on posterior wing margin midlength and CuA_2_ terminating on posterior wing margin near the basal one-third of wing length; CuP simple, terminating on posterior wing margin near the basal one-fourth of wing length; and A simple, not connected with the posterior wing margin.

Genus *Gnethoda* Cui and Engel, 2020.

Type species. *Gnethoda symmetrica* Cui and Engel, 2020.

*Gnethoda lata* Liu, Shi, Ren and Yang sp. nov.

urn:lsid:zoobank.org:act:97EFB9D6-CF31-423C-A31E-ECBB5867F340

Figure 2.

Material. Holotype: CNU-EMB-MA2019022.

Etymology. The specific name is taken from the Latin *lata* (meaning “broad”) and refers to the broad pronotum. The gender is feminine.

Locality and horizon. Hukawng Valley, Kachin State, northern Myanmar; lowermost Cenomanian, Upper Cretaceous.

Diagnosis. Head length slightly longer than width, with ocelli present, dorsal side with Y-shaped sutura and postocular carinae; ventral bridge absent; metabasitarsus with two plantulae; cerci nearly symmetrical, consist of two segments. Forewing with MA and CuA simple; three r-rs crossveins, one rs+ma-mp crossvein, and two mp-cua crossveins in the midlength of the wing.

**Figure 2:**
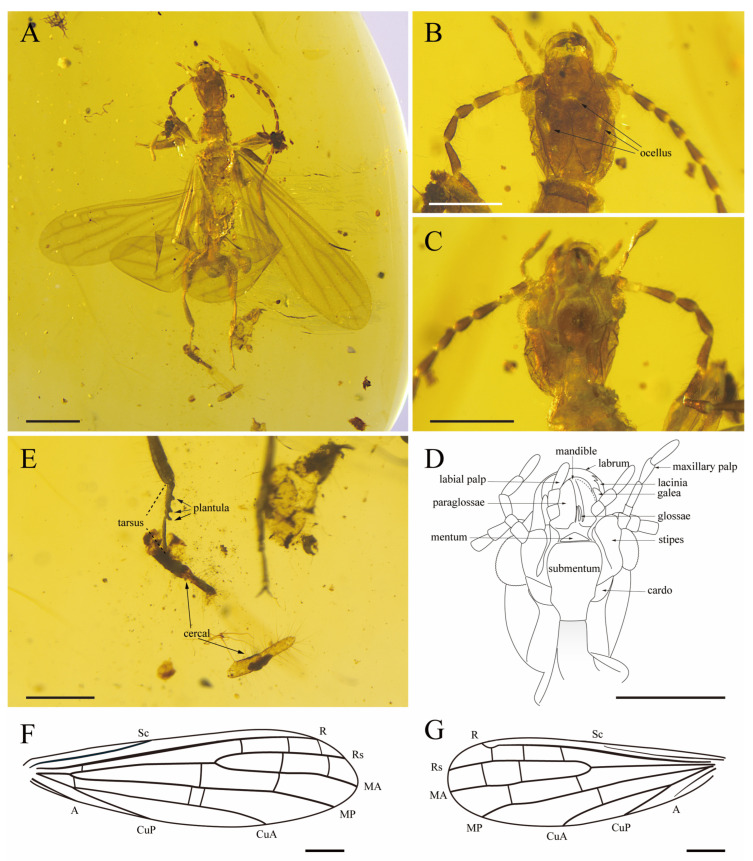
*Gnethoda lata* sp. nov., holotype (CNU-EMB-MA2019022). (**A**) Habitus, dorsal view; (**B**) photograph of head in dorsal view and (**C**) ventral view; (**D**) line drawing of head in ventral view; (**E**) tarsus of hind leg and broken cerci, dorsal view; (**F**) line drawing of right forewing; (**G**) line drawing of left hind wing. Scale bars: (**A**) 1 mm, (**B**–**G**) 0.5 mm.

Description. Male. Integument generally dark brown (Figure 2). Head oval-shaped, length (to apex of labrum) ca. 0.97 mm, width (just posterior to compound eyes) ca. 0.74 mm; compound eyes well-developed and prominent; a pair of lateral ocelli located below the inner margin of compound eyes, median dorsal ocellus positioned in the middle between the two compound eyes (Figure 2B); ventral bridge absent (Figure 2C,D); labial palpus with three palpomeres, third palpomere longest; maxillary palpus with five palpomeres, first to third palpomeres of equal length, apical palpomere longest; antenna preserved well, total length ca. 3.54 mm with 14 flagellomeres, each flagellomere with intensive, long setae, scape longer than pedicel, and they are both longer than wide, first flagellomere length about three times as long as width, second to third flagellomeres length about two times as long as width, fourth through fourteenth flagellomeres length about 3.5 times as long as width. Pronotum length ca. 0.52 mm, width ca. 0.62 mm; anterolateral angles rounded; anterior margin weakly concave; posterior margin rounded; division into pro- and metazones distinct. Probasitarsus widened, length about three times as long as width (about 0.54 mm × 0.18 mm). Hind femora moderately widened; metabasitarsus with two plantulae (Figure 2E): located at the middle and apex of the segment; second tarsomere with one plantula. All claws simple and symmetrical, arolia absent. The abdominal structure not intact, but cerci visible. Cerci (Figure 2E) nearly symmetrical, consisting of two cylindrical segments with slender setae. LC_1_ width: ca. 0.12 mm; LC_2_ length: ca. 0.28 mm, width: ca. 0.08 mm; RC_1_ width ca. 0.11 mm; and RC_2_ length: ca. 0.26 mm, width: ca. 0.06 mm.

Forewing (Figure 2F): length ca. 4.30 mm, width ca. 1.23 mm, wings hyaline; Sc terminating near the basal two-fifths of the wing length, not connected with the anterior wing margin; R strong, connected with the anterior wing margin, with one r-rs+ma crossvein present in the basal one-sixth of the wing; Rs and MA separating at the apical two-fifths of the wing length; Rs simple, terminating on anterior wing margin, with three r-rs crossveins present in apical half of wing; MA simple, terminating on wing apex, with two rs-ma crossveins; MP simple, terminating on posterior wing margin, with one ma-mp crossvein in apical one-fourth of wing and one rs+ma-mp crossvein in one half of wing; CuA simple, terminating on posterior wing margin in the apical one-third of wing length, with three mp-cua crossveins—one present at anal area near the fork of CuA and CuP, and the others present in midlength of wing; CuP simple, terminating on posterior wing margin in the basal one-third of wing length; A short and simple, connected with posterior wing margin.

Hind wing (Figure 2G): length ca. 3.60 mm, width ca. 1.16 mm, wings hyaline; Sc terminating on the wing margin near the basal two-fifths of the wing length, not connected with anterior wing margin; R strong, connected with anterior wing margin; Rs simple, terminating on the anterior wing apex, with three r-rs crossveins present in the apical half of wing; MA simple, terminating at wing apex, with two rs-ma crossveins; MP simple, terminating on posterior wing margin, with two ma-mp crossveins; CuA simple, terminating on posterior wing margin apical one-half of the wing length, with one mp-cua crossvein present in midlength of wing; CuP simple, terminating on posterior wing margin near the basal one-third of wing length; A simple, not connected with posterior wing margin.

Remarks. This species can be assigned to the *Genethoda* Cui and Engel, 2020, due to the following features: (1) cylindrical cerci with two segments; (2) MA, MP, CuA and CuP all not forked; (3) ma-mp crossveins present; (4) CuA elongate, terminating on posterior wing margin apical one-third of wing length.

The new species are distinguished from other species by the number of r-rs crossveins and rs+ma-mp crossveins in the forewing: (1) *G. lata* sp. nov. with three r-rs crossveins; *Genethoda symmetrica* Cui and Engel, 2020, *Genethoda ancyla* Cui and Engel, 2020, and *Genethoda putshkovi* Anisyutkin and Perkovsky, 2022, with four r-rs crossveins; and *Genethoda odntophora* Lai, Yang and Zhang, 2022, with four to five r-rs crossveins. (2) *G. lata* sp. nov. with one rs+ma-mp crossvein, but no rs+ma-mp crossvein present in other species of the genus.

Due to the intact state of preservation of the specimen, the ocelli and Y-shaped sulcus are determined. *G. lata* sp. nov. could be clearly observed to have three ocelli, which echo the structure of the suspected ocelli in *G*. *putshkovi* Anisyutkin and Perkovsky, 2022. We adhere to the diagnosis of genus given by Cui et al. [12] with a single correction: ocelli absent or present.

Family Scelembiidae Ross, 2001.

Subfamily Sorellembiinae Engel and Grimaldi, 2016.

Genus *Parasorellembia* Anisyutkin and Perkovsky, 2022.

Type species. *Parasorellembia groehni* Anisyutkin and Perkovsky, 2022.

*Parasorellembia hamata* Liu, Shi, Ren and Yang sp. nov.

urn:lsid:zoobank.org:act:F73356BB-94C3-4870-BC68-EC998E177CDF

Figure 3.

Material. Holotype: CNU-EMB-MA2019023.

Etymology. The specific name is derived from the Latin *hamata* (meaning “hooked”), in reference to HP with an upward hook in apical. The gender is feminine.

Locality and horizon. Hukawng Valley, Kachin State, northern Myanmar; lower-most Cenomanian, Upper Cretaceous.

Diagnosis. Ocelli present; terminalia strongly asymmetrical; the left cercus fused and curved, right cercus robust, apically, with a curved, long and thin outgrowth; H large, and HP with an upward hook in the apical; forewing with MA unforked, four r-rs crossveins, one mp-cua crossvein and two cu-a crossveins.

**Figure 3:**
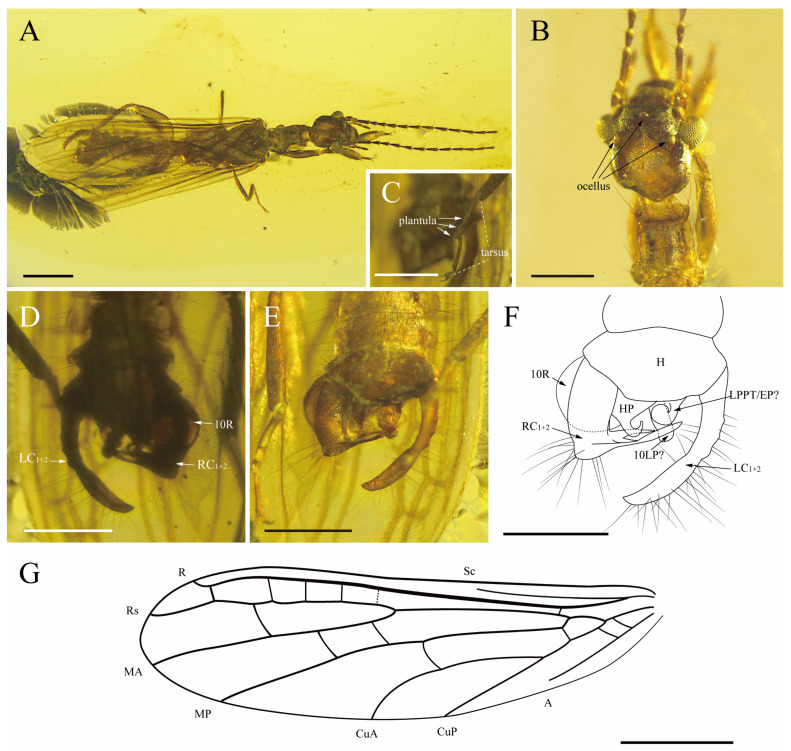
*Parasorellembia hamata* sp. nov., holotype (CNU-EMB-MA2019023). (**A**) Habitus, dorsal view; (**B**) photograph of head in dorsal view and (**C**) tarsus of left hind leg in ventral view; (**D**) photograph of male genitalia in dorsal view and (**E**) ventral view; (**F**) line drawing of male genitalia in ventral view; (**G**) line drawing of left forewing. Scale bars: (**A**,**G**) 1 mm, (**B**–**F**) 0.5 mm.

Description. Male. Integument brown overall (Figure 3A). Body total length (excluding wings, antennae, and cerci) ca. 5.60 mm. Head oval-shaped, length (to apex of labrum) ca. 1.02 mm, width (just posterior to compound eyes) ca. 0.66 mm, vertex pale; compound eyes well developed, prominent, a pair of lateral ocelli located at the inner margin of compound eyes, median dorsal ocellus located behind epistomal suture (Figure 3B); anterior edge of the labrum is rounded, labial palpus with three palpomeres—equal lengths for first and second palpomeres, third palpomere longest; maxillary palpus with five palpomeres, first to second palpomeres of equal length, third and fourth palpomeres longer than before, apical palpomere longest; antenna preserved well, total length ca. 2.90 mm with 12 flagellomeres—each flagellomere with intensive, long setae scape and pedicel of equal length, first flagellomere length about four times as long as width, second to fourth flagellomeres length about two times as long as width, and the fifth through twelfth flagellomeres length about 3.5 times as long as width. Pronotum length ca. 0.55 mm, width ca. 0.46 mm; anterolateral angles orthogonal; anterior margin weakly concave; posterior margin orthogonal; division into pro- and metazones distinct. Probasitarsus widened, length about 2.5 times as long as width (about 0.53 mm × 0.20 mm). Hind femora moderately widened, metabasitarsus with two plantulae (Figure 3C) located at the middle and apex of the segment; second tarsomere with one plantula. All claws simple and symmetrical, arolia absent. The ventral and dorsal plates of the abdomen cuticularized, and the margin of abdomen distributed with elongated setae. Terminalia (Figure 3D–F) asymmetrical; tenth abdominal tergum divided into left and right hemitergites (but the boundary between the two hemitergites indistinguishable due to the occlusion of the wings); a slender sclerite plate in the middle of the tenth abdominal tergite (may be 10LP); a large sclerite (10R) nearly cover over the right cercus; an upward hook at the end of HP; the left cercus fused and curved, length ca. 0.85 mm, width ca. 0.10 mm; right cercus robust, apically with a curved, long and thin outgrowth.

Forewing (Figure 3G): length ca. 4.80 mm, width ca. 1.39 mm, wings hyaline; Sc terminating near the basal two-fifths of wing length, not connected with anterior wing margin; R strong, connected with anterior wing margin, with one r-rs+ma crossvein present in basal one-fifth of wing; Rs and MA separating nearly one half of wing length; Rs simple, terminating on anterior wing margin, with four or five r-rs crossveins present in apical half of wing; MA simple, terminating on wing apex, with one rs-ma crossvein; MP simple, terminating on posterior wing margin, with two ma-mp crossveins; CuA simple, terminating on the posterior wing margin in the apical two-fifths of wing length, with one mp-cua crossvein and one mp-cu crossvein present in basal one-fifth of the wing; CuP simple, terminating on the posterior wing margin basal three-sevenths of the wing length; and A simple, not connected with the posterior wing margin, with two cu-a crossveins present in the basal of the wing.

Remarks. This species can be assigned to *Parasorellembia* Anisyutkin and Perkovsky, 2022, because of the following characteristics: (1) ocelli present; (2) forewing with MA not forked (MA forked in *Sorellembia* Engel and Grimaldi, 2006, *Kumarembia* Engel and Grimaldi, 2011 and *Multivena* Lai, Yang and Zhang, 2022); (3) terminalia strongly asymmetrical; and (4) left cercus with single elongated segment (left cercus with two segments in *Sorellembia* Engel and Grimaldi, 2006 and *Kumarembia* Engel and Grimaldi, 2011), right cercus robust (considered to be 10RP in *P. groehni* Anisyutkin and Perkovsky, 2022).

The new species can be distinguished from *P*. *groehni* Anisyutkin and Perkovsky, 2022, by following characters: (1) forewing with four r-rs crossveins (forewing with three r-rs crossveins in *P*. *groehni*); (2) forewing with one mp-cua crossvein (forewing with no mp-cua crossvein in *P*. *groehni*); (3) forewing with two cu-a crossveins (forewing with no cu-a crossvein in *P*. *groehni*); and (4) HP with an upward hook in the apical area (HP slightly curved and sickle-shaped in *P*. *groehni*).

## 4. Discussion

Until now, 12 fossil webspinner species have been reported from the Cretaceous, of which seven are grouped in Clothodidae, and all of these are found in the Hukawng Valley of northern Myanmar. It is inferred that Clothodidae exhibited high species richness and diversity in the Upper Cretaceous.

In the fossil record of Clothodidae, the tenth abdominal tergum neither divided into hemitergites, nor was it cleft or medially emarginated. However, extant species mostly medially emarginate, such as *Clothoda longicauda* Ross, 1987; even the tenth abdominal tergum is almost completely divided into hemitergites, such as *Clothoda aequicercata* (Enderlein, 1912) [37]. Therefore, it is speculated that the non-cracking of the tenth abdominal tergum may be the plesiomorphy of Clothodidae, and the characteristics of the cracking have gradually evolved.

The absence of a right cercus is an important feature of the subfamily Sorellembiinae, but the first reported species, *Sorellembia estherae* Engel and Grimaldi, 2006, has incompletely preserved terminalia [22]. *P*. *groehni* Anisyutkin and Perkovsky, 2022, leaves the possibility of explaining the structure as a large right cercus because the right outgrowth is separated from the tenth tergite [14]. In *P*. *hamata* sp. nov., a hemitergite is present over the “right outgrowth”; it should be the right cercus. So, it is possible that the Sorellembiinae have cerci, and due to previous specimens not being well-preserved, this makes them undetectable.

**Figure 4:**
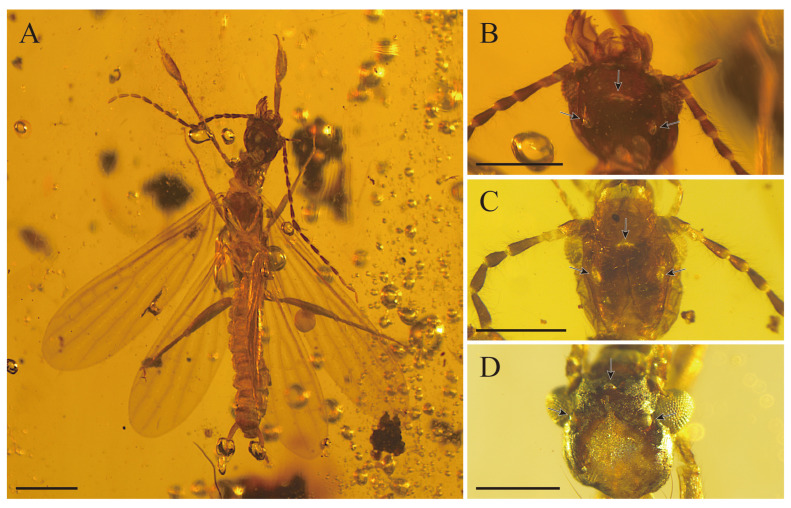
(**A**) *Gnethoda odontophora* Lai, Yang and Zhang, 2022, habitus, dorsal view; (**B**) head of *Gnethoda odontophora* Lai, Yang and Zhang, 2022, showing the ocelli; (**C**) head of *Gnethoda lata* sp. nov., showing the ocelli; (**D**) head of *Parasorellembia hamata* sp. nov., showing the ocelli. Scale bars: (**A**) 1 mm, (**B**–**D**) 0.5 mm.

All extant webspinners have no ocelli [2,22]. However, Anisyutkin and Perkovsky first described the presence of ocelli in *P. groehni* Anisyutkin and Perkovsky, 2022 [14]. Subsequently, in this study, ocelli are clearly observed in *P. hamata* sp. nov. and *G. lata* sp. nov. Additionally, after re-examining *Gnethoda odontuphora* Lai, Yang and Zhang, 2022 (Figure 4), and checking the figures of *Atmetoclothoda orthotenes* Engel and Huang, 2016 and *Litoclostes delicatus* Engel and Huang, 2016 [11], we also detected the presence of ocelli. It is inferred that a certain number of webspinners had ocelli in the Cretaceous. The discovery of this structure can provide materials for the evolution of ocelli in embiids and may provide an important basis for further exploring its adaptation to the habitat.

## 5. Conclusions

One new genus and three new species are described from the Upper Cretaceous. *Ocrognethoda olivea* gen. et sp. nov. and *Gnethoda lata* sp. nov. are classified in Clothodidae and *Parasorellembia hamata* sp. nov. is classified in Scelembiidae. The description of the new species increases the diversity of embiids in the Mesozoic. The catalogue of fossil members indicates that the Cretaceous webspinners have the highest species diversity, especially Clothodidae. Based on the comparison of extant and extinct species of this family, it is suggested that the non-cracking of the male tenth abdominal tergum may be a plesiomorphy. Moreover, an increasing number of Cretaceous webspinner species with ocelli are being described, providing material for understanding their evolution and adaptation.

## Figures and Tables

**Table 1:** Described fossil webspinners (updated from Engel et al. [21]).

Current Name	Geological Age	Distribution	Reference
Alexarasniidae			
*Alexarasnia limbata* Aristov, 2017	Permian (Capitanian)	Russia	[5]
*Alexarasnia rossica* Gorochov, 2011	Permian (Capitanian)	Russia	[6]
*Madygembia rasnitsyni* Aristov and Storozhenko, 2021	Middle–Upper Triassic (Ladinian–Carnian)	Kyrgyzstan	[7]
*Nestorembia novojilovi* Shcherbakov, 2015	Middle–Upper Triassic (Ladinian–Carnian)	Kyrgyzstan	[8]
*Nestorembia shcherbakovi* Aristov, 2017	Middle–Upper Triassic (Ladinian–Carnian)	Kyrgyzstan	[5]
*Nikloembia kusnezovi* Shcherbakov, 2015	Permian (Capitanian)	Russia	[8]
*Palaeomesorthopteron pullum* Aristov, Grauvogel-Stamm and Marchal-Papier, 2011	Triassic (Anisian)	France	[7,9]
Anisembiidae			
*Glyphembia amberica* Ross, 2003	Miocene (Burdigalian)	Dominican Republic	[10]
*Glyphembia vetehae* (Szumik, 1998)	Miocene (Burdigalian)	Dominican Republic	[10]
*Poinarembia rota* Ross, 2003	Miocene (Burdigalian)	Dominican Republic	[10]
Clothodidae			
*Atmetoclothoda orthotenes* Engel and Huang, 2016	Cretaceous (Cenomanian)	Myanmar	[11]
*Gnethoda ancyla* Cui and Engel, 2020	Cretaceous (Cenomanian)	Myanmar	[12]
*Gnethoda lata* Liu, Shi, Ren and Yang sp. nov.	Cretaceous (Cenomanian)	Myanmar	This study
*Gnethoda odontophora* Lai, Yang and Zhang, 2022	Cretaceous (Cenomanian)	Myanmar	[13]
*Gnethoda putshkovi* Anisyutkin and Perkovsky, 2022	Cretaceous (Cenomanian)	Myanmar	[14]
*Gnethoda symmetrica* Cui and Engel, 2020	Cretaceous (Cenomanian)	Myanmar	[12]
*Henoclothoda simplex* Cui and Engel, 2020	Cretaceous (Cenomanian)	Myanmar	[12]
*Ocrognethoda olivea* Liu, Shi, Ren and Yang gen. et sp. nov.	Cretaceous (Cenomanian)	Myanmar	This study
*Perissoclothoda myrrhokaris* Chen and Zhang, 2023	Cretaceous (Cenomanian)	Myanmar	[15]
Embiidae			
*Electroembia antiqua* (Pictet, 1854)	Eocene (Lutetian)	Baltic	[16]
*Galloembia raholai* Falières, Engel and Nel 2021	Eocene (Ypresian)	France	[17]
*Lithembia florissantensis* (Cockerell, 1908)	Eocene–Oligocene	Colorado	[18]
Notoligotomidae			
*Burmitembia venosa* Cockerell, 1919	Cretaceous (Albian)	Myanmar	[19]
Oligotomidae			
*Litoclostes delicatus* Engel and Huang, 2016	Cretaceous (Cenomanian)	Myanmar	[8]
Rasnalexiidae			
*Rasnalexia rasnitsyni* Gorochov, 2021	Middle–Upper Triassic (Ladinian–Carnian)	Kyrgyzstan	[20]
Scelembiidae			
*Kumarembia hurleyi* Engel and Grimaldi, 2011	Eocene (Ypresian)	India	[21]
*Multivena curvivena* Lai, Yang and Zhang, 2022	Cretaceous (Cenomanian)	Myanmar	[13]
*Parasorellembia groehni* Anisyutkin and Perkovsky, 2022	Cretaceous (Cenomanian)	Myanmar	[14]
*Parasorellembia hamata* Liu, Shi, Ren and Yang sp. nov.	Cretaceous (Cenomanian)	Myanmar	This study
*Sorellembia estherae* Engel and Grimaldi, 2006	Cretaceous (Albian)	Myanmar	[22]
Teratembiidae			
*Oligembia vetusta* Szumik, 1994	Miocene (Burdigalian)	Dominican Republic	[23]

## Data Availability

This published work and the nomenclatural acts it contains have been registered in ZooBank, the online registration system for the ICZN (International Code of Zoological Nomenclature). The LSID (Life Science Identifier) for this publication is urn:lsid:zoobank.org:pub:83E12FAF-1344-4E23-88C2-5C876F673E72.
